# Stilbenes in Red Wine: Formation and Biological Potential of Resveratrol and Piceid Dimers

**DOI:** 10.3390/molecules29246067

**Published:** 2024-12-23

**Authors:** Ayoub Jaa, Patricia Homobono Brito de Moura, Josep Valls-Fonayet, Grégory Da Costa, María Begoña Ruiz-Larrea, Stéphanie Krisa, José Ignacio Ruiz-Sanz, Tristan Richard

**Affiliations:** 1 Bordeaux INP, INRAE, Bordeaux Sciences Agro, OENO, UMR 1366, ISVV, University of Bordeaux, F-33140 Villenave d’Ornon, France; ayoub.jaa@u-bordeaux.fr (A.J.); patricia.homobono-brito-de-moura@inrae.fr (P.H.B.d.M.); josep.valls-fonayet@u-bordeaux.fr (J.V.-F.); gregory.da-costa@u-bordeaux.fr (G.D.C.); stephanie.krisa@u-bordeaux.fr (S.K.); 2 Free Radicals and Oxidative Stress (FROS) Research Group, Department of Physiology, Medicine and Nursing School, University of the Basque Country UPV/EHU, 48940 Leioa, Spain; mbego.ruizlarrea@ehu.eus (M.B.R.-L.);

**Keywords:** δ-viniferin, δ-viniferin-diglucoside, polymerization, anti-inflammatory effects

## Abstract

Resveratrol and its glucoside, piceid, are the primary stilbenes present in wine. These compounds are well known for their pharmaceutical properties. However, these compounds can undergo chemical transformations in wines, such as polymerization in the presence of metallic reagents. This study investigates the oxidative coupling of resveratrol and piceid to form dimers, including δ-viniferin and δ-viniferin-diglucoside. These dimers were synthesized using silver acetate. The formation of these stilbenes was monitored in wine model solutions and red wines. The results indicated that resveratrol and piceid underwent transformation during heat treatment, forming their respective dimers. The polymerization of both compounds is temperature-dependent, with higher conversion rates at elevated temperatures. Notably, piceid was more reactive than resveratrol in wine. Finally, the anti-inflammatory effects of these compounds were evaluated on the RAW 264.7 macrophage cell line.

## 1. Introduction

Stilbenes are a family of phenolic compounds present in many plants, including grapevine [1,2]. These compounds have many potential biological properties in terms of both plant health [3] and human health [2,4]. Resveratrol (*trans*-3,5,4′-trihydroxystilbene) is the best-known stilbene for its pharmaceutical potential [5,6]. Along with its glucoside, piceid (*trans*-3,5,4′-trihydroxystilbene-3-*O*-*β*-D-glucoside), also named polydatin, resveratrol is naturally present in wine [7]. Piceid is typically the most abundant stilbene in red wine, with concentrations reaching a few milligrams per liter. Red wine is considered as the primary dietary source of resveratrol and piceid in Mediterranean countries [8].

Resveratrol is a polyphenol composed of two aromatic rings linked by a carbon–carbon double bond. Under specific conditions, such as exposure to metallic catalysts, resveratrol can undergo polymerization to form complex compounds such as dimers and tetramers [9]. This polymerization process could occur during winemaking and aging [10], influencing key aspects of wine, such as color, flavor, and antioxidant capacity. While resveratrol is known for its antioxidant properties, the polymerized forms may contribute to the overall health benefit of wine’s polyphenolic content [11].

This study used an oxidative coupling reaction protocol to obtain resveratrol and piceid dimers in ethanol using silver acetate (AgOAc). Our study aimed to investigate the presence of these stilbenes in the wine. The resulting compounds were identified by MS and NMR analysis. Following their identification, we studied the formation of these stilbenes in wine model solutions and red wine after heat treatment using a LC-QqQ-MS method. We also assessed their abilities to prevent lipopolysaccharide (LPS)-induced NO production in the RAW 264.7 macrophage cell line, comparing their efficacy to resveratrol.

## 2. Results and Discussion

### 2.1. Synthesis of δ-Viniferin and δ-Viniferin Diglucoside

**Figure 1:**
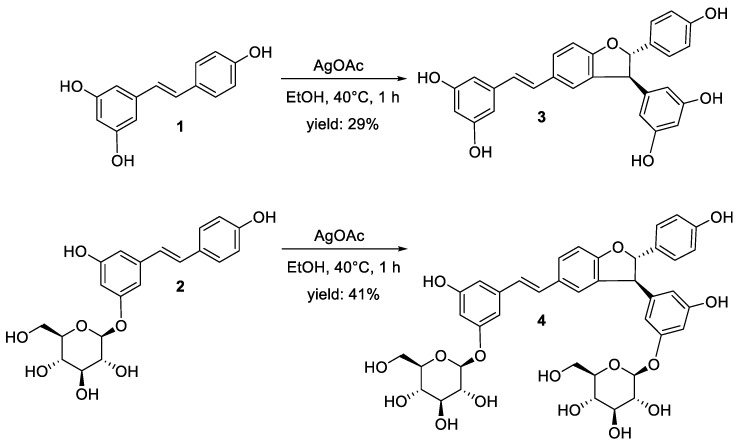
Synthetic pathways for δ-viniferin (**3**) and δ-viniferin diglucoside (**4**) from resveratrol (**1**) and piceid (**2**), respectively.

δ-viniferin (**3**) and δ-viniferin diglucoside (**4**) standards (Figure 1) are not commercially available. These compounds were synthesized from resveratrol (**1**) and piceid (**2**) via oxidative coupling, following our previously reported protocol for resveratrol [10]. The oxidative coupling of resveratrol, catalyzed by silver acetate, begins with hydrogen abstraction from a phenolic hydroxyl group (one-electron oxidation), followed by regioselective coupling and intramolecular cyclization [9]. Resveratrol and piceid dimers were produced by dissolving in ethanol and exposing them to silver acetate (AgOAc) for 1 h at 40 °C (Figure 1). The main reaction products were fully characterized by NMR spectroscopy and LC-MS. ^1^H NMR spectra of δ-viniferin (**3**) and δ-viniferin diglucoside (**4**) were provided as Supplementary Materials (Figures S1 and S2, respectively). Consistent with our previous results concerning resveratrol [10], δ-viniferin (a C3-C8′ regioisomer) and δ-viniferin diglucoside were the predominant dimers formed from resveratrol and piceid, respectively, along with minor dimer species [10,12].

### 2.2. Monitoring Resveratrol and Piceid Stability

A previous study demonstrated that resveratrol can undergo oxidative coupling in wine following heat treatment [10]. Despite regulated metal levels, trace metals in wine can still promote oxidative coupling. This study aimed to explore the conditions under which these reactions occur in wine subjected to moderate heat treatment. Given that the two primary forms of stilbenes in wine are piceid followed by resveratrol [7], we examined for the first time the oxidative coupling of both compounds in wine-like matrices containing metal salts.

**Figure 2:**
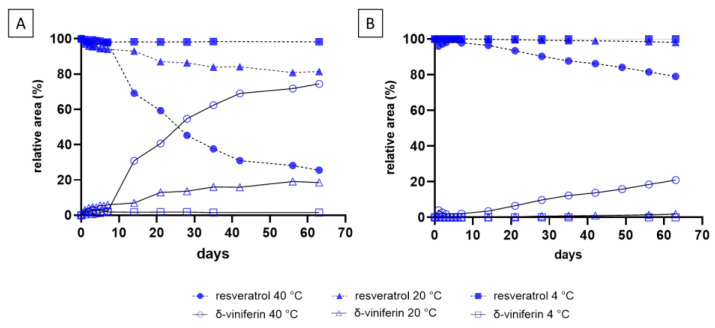
Resveratrol (100 mg/L) conversion kinetics at 4 °C, 20 °C, and 40 °C in SLW1 (**A**) and SLW2 (**B**), monitored using HPLC-DAD (λ = 306 nm) over 63 days.

Figure 2 illustrates the dimerization kinetics of resveratrol in wine-like solutions (SLW1: 12% EtOH, and SLW2: EtOH 12% and 0.1 g/L tartaric acid). The conversion of resveratrol (100 mg/L) to δ-viniferin was monitored at 4 °C, 20 °C, and 40 °C over 63 days.

The results showed that this reaction is temperature-dependent, with higher conversions obtained at 40 °C of resveratrol to δ-viniferin. The reaction in SLW2 occurs in an acidic medium due tartaric acid (pKa_1_ 3.1 and pKa_2_ 4.2), a typical acid in wines [13]. Resveratrol has pKa values of 8.8 (pKa_1_) and 9.8 (pKa_2_) [14]. In one-electron oxidation reactions, where a hydrogen atom is lost, the group from which the hydrogen is removed is usually the one that is more acidic (lower pKa). This observation may explain the lower conversion of resveratrol into δ-viniferin in the acidic medium (Figure 2B).

**Figure 3:**
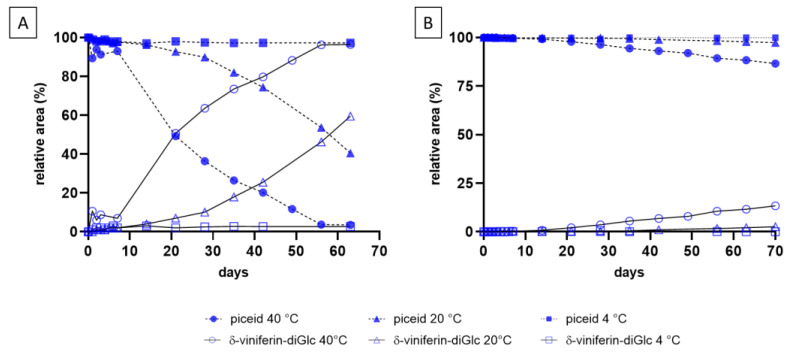
Piceid (100 mg/L) conversion kinetics at 4 °C, 20 °C, and 40 °C in SLW1 (**A**) and SLW2 (**B**) monitored using HPLC-DAD (λ = 306 nm) over 63 days (δ-viniferin-diGlc = δ-viniferin diglucoside).

Similar experiments were conducted with piceid, and the results, shown in Figure 3, confirmed conversion into δ-viniferin-diglucoside under both solutions. As with resveratrol, the dimerization was temperature-dependent, and it occurred more rapidly than resveratrol at 40 °C (Figure 2A and Figure 3A). The acidic medium (Figure 3B) also resulted in a slower conversion rate for piceid.

The same reaction behavior at a concentration of 10 mg/L of the stilbenes was also monitored over 63 days at 40 °C and showed a similar kinetic profile, with higher conversions in the SLW1 solution compared to SLW2, as shown in Figure S3 (Supplementary Materials). These results indicate that both temperature and pH influence the stability of resveratrol and its glucoside in a hydroalcoholic medium containing metal salts.

### 2.3. Dimerization of Resveratrol and Piceid in Red Wines

Considering the polymerization products obtained in wine-like solutions, we investigated in the present work the possibility of producing the same derivatives in red wines without adding reagents. Our experiments with model solutions demonstrated that the dimerization process is slow and significantly influenced by external conditions, such as temperature and the reaction medium. Three red wines were stored at 40 °C in the dark for 63 days. The compounds were quantified using LC-QqQ-MS before and after heat treatment on wine samples that had been concentrated 20-fold. Calibration ranges, limits of detection (LOD), and quantification (LOQ) for the analytical method are presented in Table 1, with additional validation parameters in Supplementary Materials (Table S1).

The concentrations of resveratrol (**1**) and piceid (**2**), as well as their oxidative coupling products δ-viniferin (**3**) and δ-viniferin-diglucoside (**4**), respectively, are shown in Table 2. In all wines, piceid levels were consistently higher than resveratrol, which is in line with previous findings [7].

Regarding resveratrol, the measured values indicate its consumption after 63 days, suggesting its transformation into other compounds. However, post-treatment analysis of the wines did not show a significant increase in δ-viniferin concentrations (Table 2). Interestingly, the δ-viniferin initially present in the wines appears to undergo further reactions during the treatment, as evidenced by wine 3. This finding underscores that resveratrol and its dimer undergo transformations over time due to heating. Although the long-term stability of resveratrol in wines has not been extensively investigated, it is well established that factors such as pH and temperature can significantly influence its stability [15,16,17]. Our findings confirm that resveratrol undergoes transformations in wine under these conditions.

For piceid, the glycosylated monomer of resveratrol, higher concentrations were observed in the wines compared to its non-glycosylated form. Although the initial concentrations exceeded the calibration range selected for this study, piceid was also found to undergo transformation due to heat, as evidenced by wine 1. Notably, we detected the presence of its dimer, δ-viniferin-diglucoside, in wine 2, making the first identification of this compound in wine. Furthermore, δ-viniferin-diglucoside was detected in all wines after the treatment, suggesting that oxidative coupling reactions do indeed occur in wine. However, the measured concentrations, ranging from 14 to 51 μg/L, suggest that additional reactions likely occur during the heating process.

Given the relatively low concentrations of resveratrol in the wines, we conducted experiments where resveratrol or piceid (5 mg/L) was added before treatment. The resulting levels of δ-viniferin (**3**) and δ-viniferin-diglucoside (**4**) after treatment are shown in Table 3. For δ-viniferin, no significant variation in concentration was observed after treatment, even in the wines where resveratrol was added. This could be due to competition between piceid and resveratrol for dimerization. Indeed, our results showed that piceid dimerizes faster than resveratrol at 40 °C in the model solutions.

**Figure 4:**
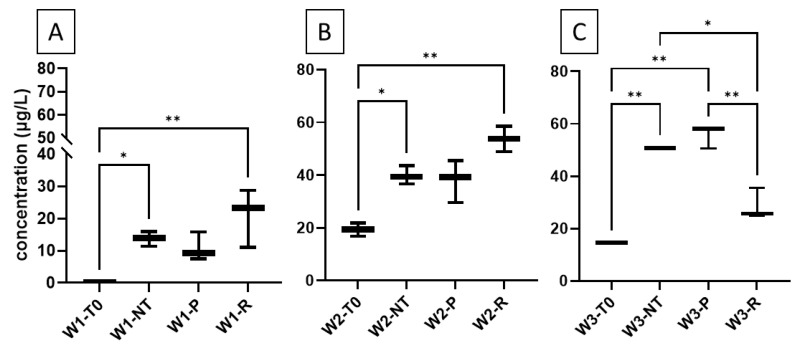
Boxplots representing the concentrations of δ-viniferin-diglucoside in wine samples ((**A**): Wine 1; (**B**): Wine 2; (**C**): Wine 3), illustrating the treatments: no treatment (NT), addition of piceid (P), and addition of resveratrol (R), alongside the initial condition (T0). Statistical differences between the NT, P, and R treatments were analyzed using Tukey’s test (*: *p* < 0.05; **: *p* < 0.01).

Regarding δ-viniferin diglucoside, we observed significantly higher levels after treatment in all the wines (Figure 4). The highest concentrations were obtained after 63 days of incubation in wine 3, particularly with the addition of piceid. However, the addition of piceid did not result in significant differences in δ-viniferin diglucoside concentrations at the end of the reaction period. This suggests that piceid addition is not a critical factor for forming its corresponding dimer. These findings indicate that the naturally occurring amounts of stilbenes in the wines are sufficient for dimer formation without adding piceid or resveratrol. The ability to form these dimers may instead depend on the presence of other reactants, such as metal ions, that can promote the dimerization process.

### 2.4. Anti-Inflammatory Activities

**Figure 5:**
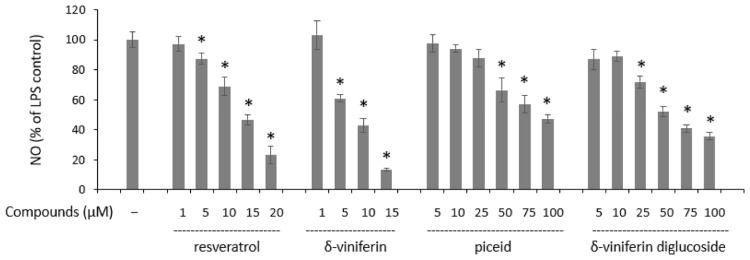
Effect of treatment with stilbene and LPS (0.1 µg/mL) on the NO production in RAW 264.7 cells. Data are expressed as a percentage of the control (cells treated with LPS alone set to 100% production), corresponding to the mean ± SEM (*n* = 4) (* *p* < 0.05).

Chronic inflammation and oxidative stress are physiological processes associated with the onset and development of various chronic diseases, including cardiovascular disease, cancer, and metabolic and neurodegenerative disorders. The anti-inflammatory activity of the produced compounds was assessed by measuring nitric oxide (NO, a marker of inflammation) in a macrophage model cell line (RAW 264.7 cells) activated with LPS. First, the cytotoxicity of the compounds was assessed. Resveratrol showed no toxicity to cells at concentrations between 1 and 20 μM, while δ-viniferin was toxic at 20 μM. Piceid and its produced dimer were non-toxic to cells up to 100 μM. The anti-inflammatory activity was then studied (Figure 5). Resveratrol and δ-viniferin had IC_50_ values below 15 μM (14.3 and 8.0 μM, respectively). At the same time, piceid and δ-viniferin-diglucoside had a more moderate inhibitory effect, with IC_50_ values of 93.0 and 54.6 μM, respectively. This shows that glycosylated stilbenes are less active than non-glycosylated (aglycone) forms. In addition, dimerization appears to increase anti-inflammatory activity.

## 3. Materials and Methods

### 3.1. Reagents

In this study, all reagents were of analytical grade and used as received without further purification. The organic solvents and acids were purchased from Fisher Scientific (Fisher Scientific, Illkirch, France). The reagents were obtained from Sigma-Aldrich (Sigma-Aldrich Chimie, Saint-Quentin-Fallavier, France) for biological assays. The silver acetate (AgOAc, purity ≥ 99%) was purchased from Acros Organics (Geel, Belgium). The ultrapure water was obtained from an Elga apparatus (High Wycombe, UK). The deuterated solvents were acquired from Eurisotop (Saint-Aubin, France). The trans-resveratrol (purity ≥ 99%) was supplied by Bulk Powders™ (Colchester, UK). Piceid was purchased from Sigma-Aldrich.

### 3.2. Production of Resveratrol and Piceid Dimers

The hemisynthesis of resveratrol and piceid derivatives was based on our previously published protocol concerning resveratrol [10]. Briefly, the oxidative coupling reaction was performed using silver acetate (1.5 equiv.) in pure ethanol. The mixture was stirred for 1 h at 40 °C, stopped by cooling at 4 °C and centrifuged at 4000 rpm for 5 min. The supernatant was collected and filtered to remove metal residues. The solvent was removed under reduced pressure. Produced dimers were isolated and identified using preparative HPLC, UHPLC-DAD, and NMR. The purity of the isolated compounds was estimated to be higher than 90%.

Data for (±)-δ-viniferin (**3**). [M-H] ^−^ 453.1334 (C_28_H_21_O_6_), Mass error = −2.21 ppm.

Data for (±)-δ-viniferin diglucoside (**4**). ^1^H NMR (600 MHz, methanol-*d*_4_): δ (ppm) 7.40 (1H, dd, *J* = 8.3 and 2.4 Hz, H6b), 7.20 (1H, brs, H2b), 7.17 (2H, d, *J* = 8.5 Hz, H2a(6a)), 7.04 (1H, d, *J* =16.4 Hz, H7b), 6.87 (1H, d, *J* = 8.3 Hz, H5b), 6.85 (1H, d, *J* =16.4 Hz, H8b), 6.79 (2H, d, *J* = 8.5 Hz, H3a(5a)), 6.77 (1H, t, *J* = 2.1 Hz, H10b), 6.60 (1H, t, *J* = 1.8 Hz, H14b), 6.49 (1H, d, *J* = 2.1 Hz, H-12a), 6.43 (1H, t, *J* = 2.1 Hz, H-12b), 6.41 (2H, d, *J* = 2.1 Hz, H-10a), 6.29 (2H, d, *J* = 1.8 Hz, H-14a), 5.41 (1H, d, *J* = 8.3 Hz, H-7a), 4.88 (1H, d, *J* = 7.5 Hz, H1″), 4.86 (1H, d, *J* = 7.2 Hz, H1′), 4.47 (1H, d, *J* = 8.3 Hz, H-8a), 3.95–3.65 (4H, H6a′(6a″) and H6b’(6b″)), 3.50-3.34 (8H, H2′ to H5′ and H2″ to H5″). [M-H]^−^ 777.2410 (C_40_H_41_O_16_^−^), Mass error = 1.29 ppm.

### 3.3. Kinetics Study of Polymerization Reaction in Wine-like Solutions

This experiment used wine-like solutions named SLW1 (12% EtOH) and SLW2 (EtOH 12%, tartaric acid 0.1 g/L). The oxidative coupling reactions of resveratrol and piceid (100 mg/L) were separately performed in wine-like solutions SLW1 and SLW2 (total volume of 5 mL) in the presence of silver acetate (AgOAc—200 mg/L) as a catalyst at 4 °C, 20 °C, and 40 °C. Additionally, the same stilbenes were reacted using both wine-like solutions (5 mL) at a concentration of 10 mg/L with AgOAc (20 mg/L) at 40 °C. Both sets of reactions were carried out for 63 days. Aliquots of 20 µL were taken at T0 and daily for 7 days, followed by weekly aliquots until day 63. All the samples were stored at −20 °C until the day of instrumental analysis for kinetic evaluation. All the samples were centrifuged at 12,000 rpm at 4 °C before analysis. The UHPLC-MS measurements were carried out using the equipment and protocols described in the previous paragraph.

Data were processed with Bruker Data Analysis 3.2 software. The areas of *t*-piceid, *t*-resveratrol, δ-viniferin diglucoside, and δ-viniferin obtained by UHPLC-DAD-MS were monitored at λ_max_ = 306 nm (maximum absorption for stilbenes) and transformed according to the equation:

$$relative\;area\;\left(\%\right)=\left(\frac{{area}^{REAG\;or\;PRO}}{{area}^{REAG}+{area}^{PRO}}\right)\times 100$$

* *REAG*: reagents (*t*-piceid or *t*-resveratrol) * *PRO*: products (δ-viniferin diglucoside or δ-viniferin).

### 3.4. Polymerization Reactions in Wines

A volume of 8 mL of three red wines was reacted under three different conditions: (1) only wines; (2) wines spiked with 5 mg/L of resveratrol; (3) wines spiked with 5 mg/L of piceid were incubated for 63 days in an orbital shaker (40 rpm) at 40 °C. Wine samples were kept away from UV light.

After the reaction, each sample (2 mL) was extracted with a Supelco C18 SPE cartridge (Merck, Darmstadt, Germany). The solution obtained was reduced in a rotary evaporator, and the residual volume was evaporated in a SpeedVac (Thermo Fisher Scientific, Waltham, MA, USA). This dried material was resuspended in 100 μL of MeOH (50%), resulting in a 20× more concentrated solution. In addition to the nine reacted samples, the unreacted wines were extracted with C18 SPE cartridges under the same conditions described above.

The quantitative analysis was carried out on a UHPLC 1290 Infinity system coupled to a triple quadrupole mass detector (6430 Triple Quad) with an electrospray ionization (ESI) interface (Agilent Technologies, Wilmington, DE, USA). All the samples were previously centrifuged at 12,000 rpm at 4 °C before being injected into the equipment. A Zorbax SB-C18 column (Agilent Technologies, Santa Clara, CA, USA) was used to separate the compounds. The mobile phases were water and acetonitrile (solvent B), both acidified with formic acid (0.1%). Elution was performed at a flow rate of 0.3 mL.min^−1^ with the following gradient: 1–10%B (4 min), 10–20%B (4–12 min), 20–30% (12–13 min); 30%B (13–16 min); 30–35%B (16–18 min); 35–50%B (18–20 min); 50–95%B (20–21 min); 95%B (21–25 min); and 95–99%B (25–28 min). The injection volume of reacted wines and standards was 5 μL. The stilbenes were quantified using Multiple Reaction Monitoring (MRM) acquisition at positive and negative ionization modes. The collision energies and MRM transitions were set as follows: resveratrol—12 eV, 229.0 → 135.0; piceid—12 eV, 389.0 → 143.0; δ-viniferin diglucoside—20 eV, 779.0 → 455.0; and δ-viniferin—12 eV, 453.0 → 359.0. The mass spectrometer was set on 3 kV capillary source voltage, 350 °C source desolvation temperature, 11 L/h (N_2_) desolvation gas flow and 3 L/h cone gas flow. A nitrogen generator from Peak Scientific (Peak Scientific Instruments Ltd., Palaiseau, France) and an argon gas bottle (Air Liquide, Paris, France) were coupled to the mass detector for gas supply. The acquisition, quantitative, and qualitative analysis were driven by the software MassHunter WorkStation (v. B.05.00 SP02, Agilent Technologies, Santa Clara, CA, USA).

The protocol of stilbenes quantification was validated considering linearity, limit of quantification and detection (LOQ and LOD), recovery, accuracy, and precision for all compounds. The calibration curves in MeOH at 5 to 400 μg/L (δ-viniferin and δ-viniferin diglucoside) and 5 to 3100 μg/L (resveratrol and piceid) were established. The linearity was considered according to the coefficients (R^2^) of quadratic equations. The limit of quantification (LOQ) and limit of detection (LOD) for each compound were calculated based on signal-to-noise ratio (S/N). Recovery, accuracy, and precision were evaluated at two concentration levels (6.5 and 0.78 mg/L). Precision (n = 4) was carried out considering the percentage of the relative standard deviation (RSD%) of stilbenes at the different concentrations. Recovery (n = 4) was determined by adding known concentrations of stilbenes to a quality control (QC) sample, which consisted of a mixture of reacted and T0 wines. The spiked samples (with stilbenes) were compared to non-spiked samples (without stilbenes) to calculate the experimental amount of stilbenes recovered relative to the amount initially added. The comparison between the obtained experimental value with known concentrations and the theoretical concentration evaluated the method accuracy (n = 4).

Statistical analyses were performed using GraphPad Prism 10.3.1 (GraphPad Software, San Diego, CA, USA). Differences among the various conditions were assessed with a one-way Analysis of Variance (ANOVA) and Tukey’s post-hoc test for pairwise comparisons. The normality of the variables was evaluated using the Shapiro–Wilk and Kolmogorov–Smirnov tests. Results with a *p*-value of less than 0.05 were considered statistically significant.

### 3.5. Biological Assays

The cell viability and nitic oxide (NO) production were assessed on RAW 264.7 cells following our previously published protocole [11]. Briefly, cell viability was measured using the MTT colorimetric assay. NO production was evaluated by measuring the nitrite content in the culture supernatant. Experiments were performed in quadruplicate and repeated at least three times. Data on NO production were analyzed using a one-way analysis of variance followed by a Dunnett’s post-test using GraphPad Prism. Statistical significance was set at *p* < 0.05.

## Figures and Tables

**Table 1:** Calibration range, limit of quantification (LOQ) and limit of detection LOD (in μg/L) for resveratrol (**1**), piceid (**2**), δ-viniferin (**3**) and δ-viniferin-di-glucoside (**4**).

Compounds	Calibration Range	LOQ	LOD
**1**	3100–9.6	9.6	2.8
**2**	3100–5.6	5.6	1.9
**3**	400–7.7	7.7	2.3
**4**	400–8.4	8.4	2.5

**Table 2:** Average concentration of resveratrol (**1**), piceid (**2**), δ-viniferin (**3**), and δ-viniferin- diglucoside (**4**) in red wines expressed in µg/L before and after treatment.

Compounds	Wine 1		Wine 2		Wine 3	
	Before	After	Before	After	Before	After
**1**	1025	19	188	ND	66	ND
**2**	OVER	365	OVER	OVER	OVER	OVER
**3**	NQ	ND	ND	NQ	38	ND
**4**	ND	14	19	40	ND	51

ND: not detected; NQ: not quantified; OVER: concentration above the calibration range (>3100 µg/L), not estimated.

**Table 3:** Average concentration (in µg/L) of δ-viniferin (**3**) and δ-viniferin-diglucoside (**4**) in red wines after treatment, with the addition of 5 mg/L of either resveratrol (+R) or piceid (+P).

Compounds	Wine 1		Wine 2		Wine 3	
	+R	+P	+R	+P	+R	+P
**3**	ND	2	ND	3	42	ND
**4**	21	11	51	34	29	56

## Data Availability

Data are contained within the article and Supplementary Materials.

## Supplementary Materials

The following supporting information can be downloaded at: https://www.mdpi.com/article/10.3390/molecules29246067/s1, Figure S1. ^1^H NMR spectra of δ-viniferin in acetone-*d*_6_; Figure S2. ^1^H NMR spectra of δ-viniferin diglucoside in methanol-*d*_6_; Figure S3. Polymerization kinetics of δ-viniferin from resveratrol (10 mg/L—A) and of δ-viniferin-diglucoside from piceid (10 mg/L—B) at 40 °C based on relative area of HPLC-DAD (λ = 306 nm) during 63 days; Table S1. Validation parameters of recovery (%), accuracy (%), and precision (RSD%) for resveratrol (**1**), piceid (**2**), δ-viniferin (**3**) and δ-viniferin-diglucoside (**4**) at 300 and 50 μg/L.

## References

[1] Rivière C., Pawlus A.D., Mérillon J.M. (2012). Natural stilbenoids: Distribution in the plant kingdom and chemotaxonomic interest in Vitaceae. Nat. Prod. Rep..

[2] Teka T., Zhang L., Ge X., Li Y., Han L., Yan X. (2022). Stilbenes: Source plants, chemistry, biosynthesis, pharmacology, application and problems related to their clinical Application-A comprehensive review. Phytochemistry.

[3] Chong J., Poutaraud A., Hugueney P. (2009). Metabolism and roles of stilbenes in plants. Plant Sci..

[4] Al-Khayri J.M., Mascarenhas R., Harish H.M., Gowda Y., Lakshmaiah V.V., Nagella P., Al-Mssallem M.Q., Alessa F.M., Almaghasla M.I., Rezk A.A.-S. (2023). Stilbenes, a versatile class of natural metabolites for inflammation—An overview. Molecules.

[5] Berman A.Y., Motechin R.A., Wiesenfeld M.Y., Holz M.K. (2017). The therapeutic potential of resveratrol: A review of clinical trials. npj Precis. Oncol..

[6] Fiod Riccio B.V., Fonseca-Santos B., Colerato Ferrari P., Chorilli M. (2020). Characteristics, Biological Properties and Analytical Methods of Trans-Resveratrol: A Review. Crit. Rev. Anal. Chem..

[7] Benbouguerra N., Hornedo-Ortega R., Garcia F., El Khawand T., Saucier C., Richard T. (2021). Stilbenes in grape berries and wine and their potential role as anti-obesity agents: A review. Trends Food Sci. Technol..

[8] Zamora-Ros R., Andres-Lacueva C., Lamuela-Raventós R.M., Berenguer T., Jakszyn P., Martínez C., Sánchez M.J., Navarro C., Chirlaque M.D., Tormo M.-J. (2008). Concentrations of resveratrol and derivatives in foods and estimation of dietary intake in a Spanish population: European Prospective Investigation into Cancer and Nutrition (EPIC)-Spain cohort. Br. J. Nutr..

[9] Velu S.S., Buniyamin I., Ching L.K., Feroz F., Noorbatcha I., Gee L.C., Awang K., Wahab I.A., Weber J.-F.F. (2008). Regio- and stereoselective biomimetic synthesis of oligostilbenoid dimers from resveratrol analogues: Influence of the solvent, oxidant, and substitution. Chem. Eur. J..

[10] El Khawand T., Valls Fonayet J., Da Costa G., Hornedo-Ortega R., Jourdes M., Franc C., de Revel G., Decendit A., Krisa S., Richard T. (2020). Resveratrol transformation in red wine after heat treatment. Food Res. Int..

[11] Beaumont P., Courtois A., Atgié C., Richard T., Krisa S. (2022). In the shadow of resveratrol: Biological activities of epsilon-viniferin. J. Physiol. Biochem..

[12] Vinet J., Flourat A.L., Peyrot C., Brunois F., Brunissen F., Allais F. (2022). Optimization and green metrics analysis of the AgOAc-mediated dimerization of piceid: Toward a high-yielding and more sustainable access to δ-viniferin and synthesis of new piceid dimers. ACS Sustain. Chem. Eng..

[13] Zhao Q., Du G., Wang S., Zhao P., Cao X., Cheng C., Liu H., Xue Y., Wang X. (2023). Investigating the role of tartaric acid in wine astringency. Food Chem..

[14] Delmas D., Aires V., Limagne E., Dutartre P., Mazué F., Ghiringhelli F., Latruffe N. (2011). Transport, stability, and biological activity of resveratrol. Ann. N. Y. Acad. Sci..

[15] Zupančič Š., Lavrič Z., Kristl J. (2015). Stability and solubility of trans-resveratrol are strongly influenced by pH and temperature. Eur. J. Pharm. Biopharm..

[16] Liazid A., Palma M., Brigui J., Barroso C.G. (2007). Investigation on phenolic compounds stability during microwave-assisted extraction. J. Chromatogr. A.

[17] Tian B., Liu J. (2020). Resveratrol: A review of plant sources, synthesis, stability, modification and food application. J. Sci. Food Agric..

