# Supplementary material for: Stilbenes in Red Wine: Formation and Biological Potential of Resveratrol and Piceid Dimers
Source: Molecules. 2024 Dec 23;29(24):6067. doi: 10.3390/molecules29246067 (PMC11678275; doi:10.3390/molecules29246067)
Supplement: Supplementary file 1 [file molecules-29-06067-s001.zip › molecules-3353097-supplementary.pdf]

Supplementary Material

Stilbenes in Red Wine: Formation and Biological Potential of Resveratrol and Piceid Dimers

Ayoub Jaa [1,2,†], Patricia Homobono Brito de Moura [1,†], Josep Valls-Fonayet [1], Grégory Da Costa [1], María Begoña Ruiz-Larrea [2], Stéphanie Krisa [1], José Ignacio Ruiz-Sanz [2] and Tristan Richard [1,*]

[1] Bordeaux INP, INRAE, Bordeaux Sciences Agro, OENO, UMR 1366, ISVV, University of Bordeaux, F-33140 Villenave d'Ornon, France
[2] Free Radicals and Oxidative Stress (FROS) Research Group, Department of Physiology, Medicine and Nursing School, University of the Basque Country UPV/EHU, 48940 Leioa, Spain
* Correspondence: tristan.richard@u-bordeaux.fr
† These authors contributed equally to this work.

Supplementary Material List

Figure S1. ^{1}H NMR spectrum of δ-viniferin in acetone-d_6.

Figure S2. ^{1}H NMR spectrum of δ-viniferin diglucoside in methanol-d_4.

Figure S3. Polymerization kinectics of δ-viniferin from resveratrol (10 mg/L - A), and of δ-viniferin-diglucoside from piceid (10 mg/L - B) at 40 °C based on relative area of HPLC-DAD (λ = 306 nm) during 63 days.

Table S1. Validation parameters of recovery (%), accuracy (%) and precision (RSD%) for resveratrol (**1**), piceid (**2**), δ-viniferin (**3**), and δ-viniferin-diglucoside (**4**) at 6.5 and 0.78 mg/L.

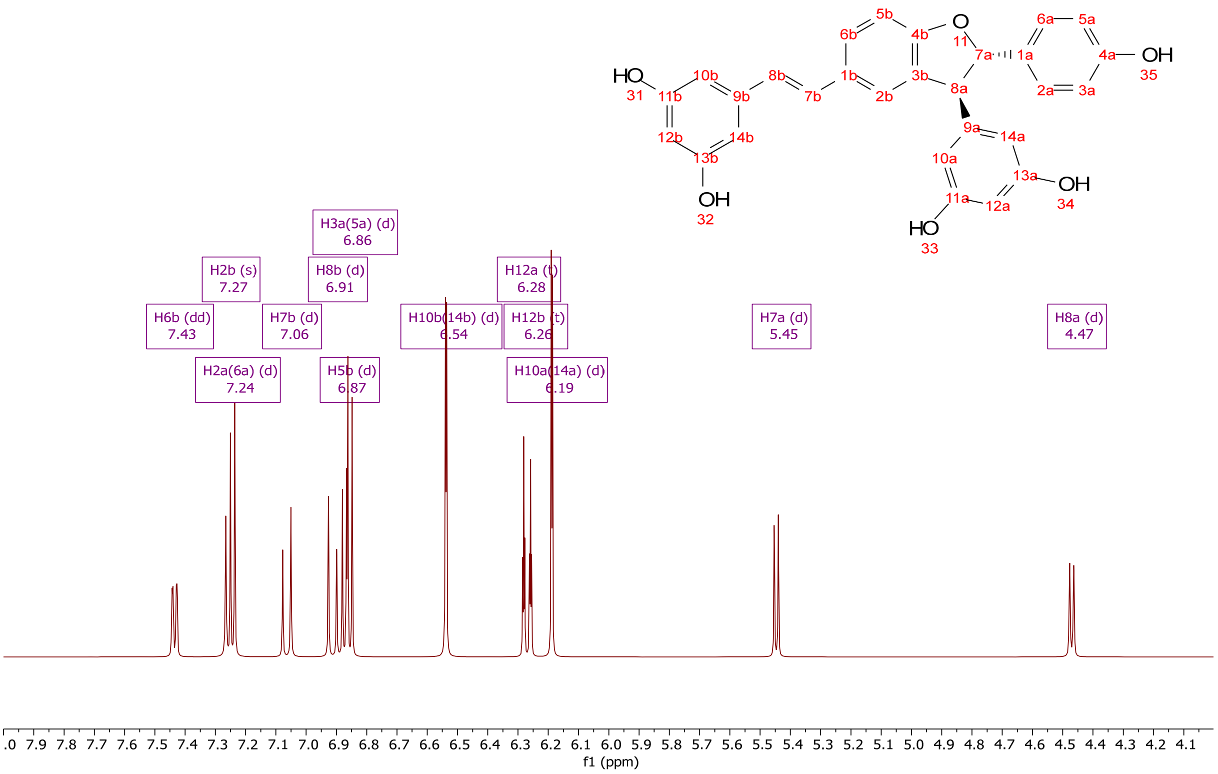

Figure S1. ^{1}H NMR spectrum of δ-viniferin in acetone-d_6.

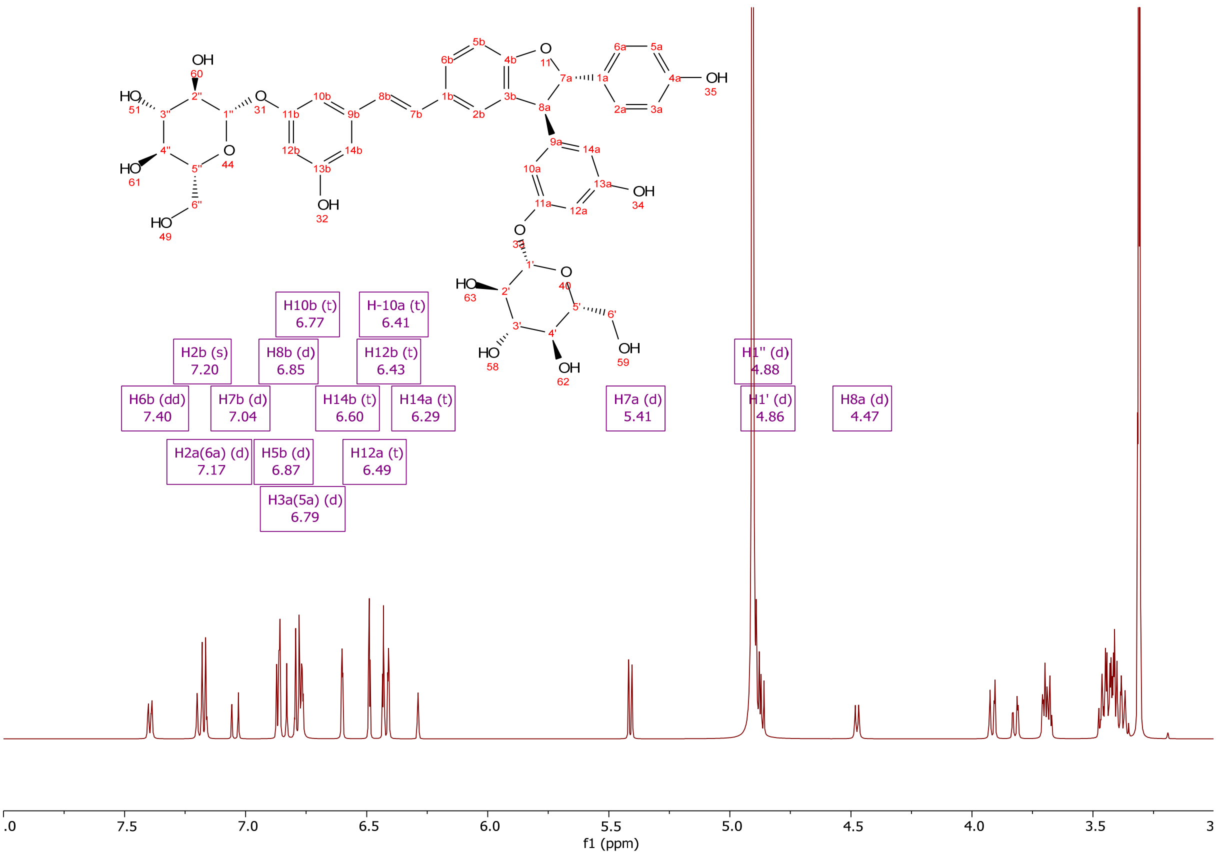

Figure S2. ^{1}H NMR spectrum of δ-viniferin diglucoside in methanol-d_4.

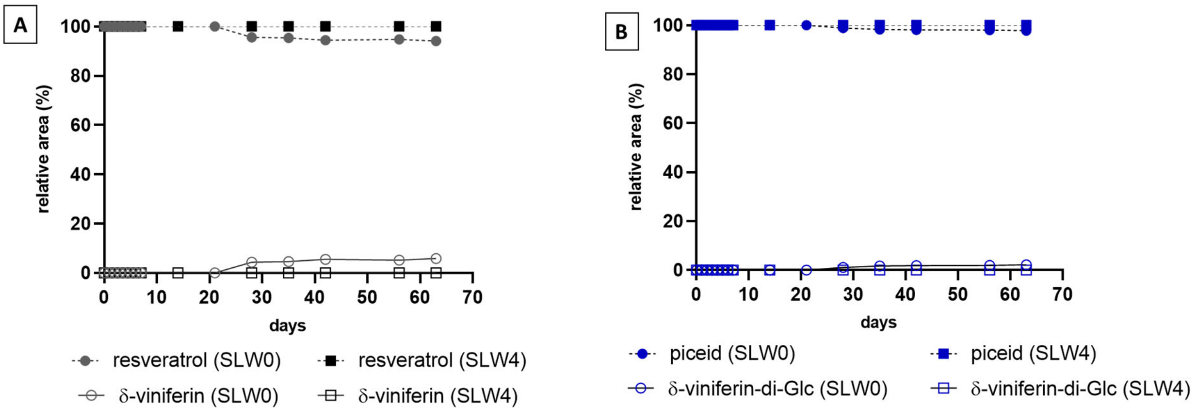

Figure S3. Polymerization kinectics of δ-viniferin from resveratrol (10 mg/L - A) and of δ-viniferin-diglucoside from piceid (10 mg/L - B) at 40 °C based on relative area of HPLC-DAD (λ = 306 nm) during 63 days.

Table S1. Validation parameters of recovery (%), accuracy (%) and precision (RSD%) for resveratrol (**1**), piceid (**2**), δ-viniferin (**3**) and δ-viniferin-diglucoside (**4**) at 300 and 50 μg/L.

	Concentration (μg/L)	Recovery (%)	Accuracy (%)	Precision (RSD%)
1	**300**	**46.12**	**83.85**	**6.22**
	**50**	**124.93**	**138.89**	**19.54**
2	**300**	**OVER**	**-**	**-**
	**50**	**OVER**	**-**	**-**
3	**300**	**47.56**	**45.57**	**12.16**
	**50**	**66.64**	**64.24**	**17.82**
4	**300**	**27.99**	**28.51**	**17.35**
	**50**	**23.42**	**43.93**	**20.99**

OVER: concentration above the calibration range (> 3100 μg/L), not estimated.
